# Insulin-Like Growth Factor Gene Polymorphisms Predict Clinical Course in Allogeneic Hematopoietic Stem Cell Transplantation

**DOI:** 10.3389/fimmu.2020.01646

**Published:** 2020-07-24

**Authors:** Maria Ebbesen, Christian Enevold, Anders Juul, Carsten Heilmann, Henrik Sengeløv, Klaus Müller

**Affiliations:** ^1^Department of Pediatrics and Adolescent Medicine, University Hospital Rigshospitalet, Copenhagen, Denmark; ^2^Institute for Inflammation Research, Center for Rheumatology and Spine Diseases, University Hospital Rigshospitalet, Copenhagen, Denmark; ^3^Department of Growth and Reproduction, University Hospital Rigshospitalet, Copenhagen, Denmark; ^4^Department of Hematology, University Hospital Rigshospitalet, Copenhagen, Denmark

**Keywords:** hematopoietic stem cell transplantation, insulin-like growth factor-1, single nucleotide polymorphisms, toxicity, systemic inflammation

## Abstract

Allogeneic hematopoietic stem cell transplantation (HSCT) is challenged by significant toxicities that are propagated by systemic inflammation caused by cytotoxic damage. Insulin-like growth factor-1 (IGF-1) is key in repair of most tissues and is to a large extent genetically determined. We investigated eight single nucleotide polymorphisms (SNPs) in the genes encoding IGF-1 and its binding protein (IGFBP3) in 543 patients undergoing HSCT to access their impact on systemic inflammation and clinical outcomes. Overall, median serum levels of both IGF-1 and IGFBP3 were found reduced from the referral until 2 years post-HSCT compared with healthy sex- and age-matched individuals, but, for individuals homozygous of the known high-producer minor allele of rs1520220 (*IGF1*), rs978458 (*IGF1*), or rs2854744 (*IGFBP3*) serum levels remained normal during the whole period. In accordance, maximum C-reactive protein levels were lower for these genotypes of *IGF1* (rs1520220: median 66 vs. 102 mg/L, *P* = 0.005 and rs978458: 53 vs. 104 mg/L, *P* < 0.001), translating into borderline significant superior survival (*P* = 0.060 for rs1520220) and reduced treatment-related mortality (*P* = 0.050 for rs978458). In conclusion, we found that three SNPs in the IGF-1 axis with known functional impact were associated with circulating IGF-1 or IGFBP-3 levels also in the setting of HSCT, and predictive of the severity of the toxic-inflammatory response during the treatment.

## Introduction

Allogeneic myeloablative hematopoietic stem cell transplantation (HSCT) has improved survival in patients with hematological cancers and in some non-malignant hematologic disorders. However, its success is challenged by significant toxicities mainly related to infections and inflammatory complications (1).

Varying degrees of systemic inflammation are seen during the first 2–3 weeks after conditioning, initiated due to damage of the mucosal barriers (2, 3). This inflammatory response has been found to be key in propagation of treatment-related complications resulting in a poor outcome (4–6). Accordingly, peak levels of C-reactive protein (CRP) in the early toxic phase after HSCT are associated with treatment-related complications, such as mucositis, acute graft-vs.-host disease (aGvHD), vascular endothelial syndromes including sinusoidal obstruction syndrome, and severe infections, and are predictive of treatment-related mortality (TRM) (4, 5, 7, 8).

Individual variation in the susceptibility to the tissue-damaging effects of the conditioning regimen is thought to explain the varying degrees of systemic inflammation and the related organ toxicities. Hence, investigation of factors controlling protection and regeneration of epithelial barriers could lead to identification of early predictive markers of patients at risk of complications as well as to new prophylactic treatment strategies.

Insulin-like growth factor-1 (IGF-1) is an endocrine and autocrine/paracrine growth factor that mediates the effects of growth hormone (GH) (9). Hepatic production seems to be the main determinant of circulating IGF-1 levels, but other sources, including vascular cells (10), gastrointestinal epithelium and intestinal smooth muscle cells (11, 12), contribute to IGF-1 production in local tissue. In the circulation, IGF-1 is predominantly bound to IGF-binding protein-3 (IGFBP-3), which is produced in the liver and promotes the effects of IGF-1 by prolonging its half-life and facilitating the transendothelial transport of IGF-1 to target cells (13). IGF-1 acts as a trophic factor for epithelial cells and also has anti-inflammatory effects through a direct action on leucocytes (14–17). It has been shown to promote regeneration of wounded epithelium, attenuate chemotherapy-induced mucositis and reduce bacterial translocation (18, 19).

In line with this, we have previously found reduced pre-transplant levels of IGF-1 and IGFBP-3 to be predictive of increased systemic inflammation, fluid retention and risk of sinusoidal obstruction syndrome in patients undergoing HSCT (20).

Although circulating levels of both IGF-1 and IGFBP-3 have been extensively studied these levels may not effectively reflect differences in the activity of these mediators in the micro-environment. However, heritability studies indicate that 40–80% of the inter-individual variations in IGF-1 and IGFBP-3 levels are determined by genetic factors (21, 22). Consequently, we found it of interest to study the ability of genetic markers of the IGF-1 axis to predict the outcome and course of HSCT.

Thus, we selected eight common single nucleotide polymorphisms (SNPs) SNPs in *IGF1* and *IGFBP3* based on validated findings of associations with alterations in circulating IGF-1 and IGFBP-3 levels and examined their association with clinical outcomes and severity of the systemic inflammatory response during the early toxic phase and clinical outcome in patients undergoing HSCT.

## Materials and Methods

### Cohort

We retrospectively included 543 patients undergoing allogeneic transplantation at University Hospital Rigshospitalet, Copenhagen, from 2004 to 2016. Inclusion criteria were age older than 0.5 years, first allogeneic HSCT, myeloablative conditioning, a matched sibling donor or unrelated donor and the use of bone marrow or peripheral blood as stem cell source.

Deposited blood samples were available from 550 of the 639 patients fulfilling the inclusion criteria and at least one SNP could be assigned for 543 patients (98.7%). Included patients did not differ significantly from non-participants in terms of sex, age, diagnosis, donor type, conditioning regimen, graft type, pre-transplant Karnofsky score or sex-mismatch. Due to the endocrinologic follow-up routine at our pediatric HSCT unit, pre-scheduled measurements of IGF-1 and IGFBP-3 were available only for the pediatric patients (*n* = 172) pre-transplant and at regular intervals post-HSCT.

### Patient Characteristics

The study included 200 children and 343 adults with a median age of 26.7 years (range 0.6–66.1 years). A total of 534 patients (98%) were Caucasians. Clinical characteristics of the patients are listed in Table 1.

**Table 1:** Patient characteristics and transplantation modalities.

**Patient characteristics**	**All patients**	**Pediatric patients with serum IGF-1/IGBP-3 levels available**
No. of patients, *n*	543	172
Age at transplantation (years), mean (range)	28.2 (0.6–66.1)	7.9 (0.6–17.6)
**Sex**		
Male/Female	315/228	105/67
**Disease at transplantation, no. of patients (%)**		
Acute myeoloid leukemia leukemia	153 (28)	29 (16)
Acute lymphoblastic leukemia	132 (24)	58 (34)
Chronic myeloid leukemia	44 (8)	3 (2)
Myelodysplastic syndrome	78 (14)	15 (9)
Other malignancies	38 (7)	13 (8)
Severe aplastic anemia	45 (8)	14 (8)
Immunodeficiency	24 (4)	17 (10)
Other non-malignant diseases	29 (5)	23 (13)
**TRANSPLANTATION DATA**		
**Donor type, no. of patients (%)**		
HLA-identical siblings	161 (30)	43 (25)
HLA-matched unrelated donors (10/10 match)	307 (57)	95 (55)
HLA-mismatched unrelated donors (9/10 or 8/10 match)	75 (14)	34 (20)
**Stem cell source, no. of patients (%)**		
Bone marrow stem cells	377 (69)	164 (95)
Peripheral blood stem cells, G-CSF mobilized	166 (31)	8 (5)
**Conditioning regimen, no. of patients (%)**		
TBI 12 Gy + CY or VP16	324 (60)	53 (31)
BU-CY	117 (22)	78 (45)
Other high-myeloablative conditioning regimens	7 (1)	7 (4)
Fludarabine-based conditioning	95 (17)	34 (20)
Sex mismatch (female donor to male recipient), no. (%)	74 (14)	28 (16)

*BU, busulphan; CY, cyclophosphamide; TBI, Total body irradiation; VP16, etoposide*.

### SNP Selection

An extensive literature search of PubMed was performed to identify peer-reviewed studies that reported SNPs associated with levels of circulating IGF-1 or IGFBP-3. A total of 78 publications were systematically screened by the following criteria: (1) a GWAS or candidate gene association study with epidemiological study design and (2) addressing a Caucasian population. Seventeen studies fulfilled these criteria.

For a SNP to be included it had to (1) be located in the *IGF1* or *IGFBP3* gene, (2) have a minor allele frequency > 5% and (3) be associated with circulating levels of the respective protein in at least one confirmatory independent study fulfilling the same study criteria. Ten SNPs fulfilled the above criteria and were genotyped (Table 2) (see Figure S1 for search terms, filtering approach and references).

**Table 2:** Genotypes of *IGF1* and *IGFBP3* and association with circulating IGF-1 and IGFBP-3.

**SNP**	**Location in gene**	**Genotype**	***N*^**a**^**	**HWE**	**IGF-1/IGFBP-3 pre-HSCT serum level**		
				***P*-value**	***n*^**b**^**	**Mean, SDS (95% CI) ^**c**^**	***P*-value^**d**^**
*IGF1*						IGF-1	
rs1520220	Intron 3	CC	361	0.4	118	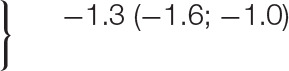	**0.002**
		CG	160		45		
		GG	21		9	0.04 (−0.7; 0.8)	
rs2946834	Downstream, 5′	CC	269	0.07	93	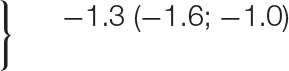	
		CT	216		64		
		TT	57		15	−0.7 (−1.5; 0.20)	0.1
rs35767	Promoter region	CC	399	1	126	−1.3 (−1.6; −1.0)	
		CT	132		44	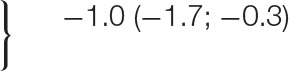	0.5
		TT	11		2		
rs7136446	Intron	TT	213	0.7	67	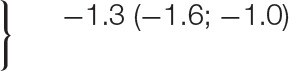	
		TC	252		83		
		CC	76		22	−0.6 (−1.2; −0.06)	0.03
rs978458 (tags rs6220)	Intron 3	GG	297	0.7	93	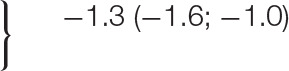	
		GA	213		64		
		AA	33		15	−0.4 (−1.0; 0.30)	**0.007**
*IGFBP3*						IGFBP-3	
rs2132571	Promoter region	GG	244	0.8	73	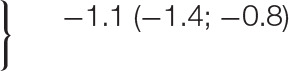	
		GA	231		76		
		AA	56		17	−1.2 (−1.7; −0.8)	0.5
rs2471551	Intron 1	GG	351	0.1	110	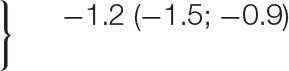	
		GC	163		51		
		CC	28		11	−0.9 (−1.6; −0.2)	0.3
rs2854746 (tags rs2854744)	Promoter region	CC	204	0.8	59	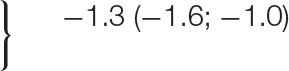	
		CG	246		84		
		GG	79		22	−0.04 (−0.8; 0.7)	**0.002**

a No of patients with certain genotype.

b No of children with certain genotype and IGF-1/IGFBP-3 serum measurement.

c SDS = Age- and sex-adjusted SD-score.

d P-value for recessive mode of inheritance; IGF-I and IGFBP-3 levels were compared between subjects who were homozygous for the minor allele and all others. rs35767 coded dominant due to MAF <15%.

*HWE, Hardy–Weinberg equilibrium; SNP, single nucleotide polymorphism. The bold of P-value is regarded statistically significant*.

### Detection of SNPs

DNA was purified from EDTA blood, collected before HSCT, with the QIAamp DNA Blood Midi Kit (QUIAGEN, Hilden, Germany). DNA was genotyped using a previously described multiplex bead-based assay-protocol (23). In brief, allele-specific primers were labeled in a primer extension using polymerase chain reaction-amplified SNP-sites as their target regions. The labeled primers were then hybridized to MagPlex-TAG beadsets for detection and counting on the Luminex platform (both Luminex Corporation, Austin, TX, USA). Positive controls obtained from the Coriell Cell Repository (Camden, New Jersey, USA) with known sequences representing the majority of possible genotypes (data not shown), as well as no-template PCR negative controls were included in all assay runs. We also included assays for the sex-specific amelogenin-gene to be able to define the sex of the patient as a quality control. All samples were carried out randomized and blinded to the technician performing the genotyping. The calling rates varied between 96.2 and 98.7, and 5% of samples were genotyped twice without discordance. One sample was excluded due to mismatch between sex according to sex determined by the amelogenin-gene and known patient sex.

### Quantification of IGF-1 and IGFBP-3 Levels

Due to the clinical routine at our institution pre-scheduled measurements of IGF-1 and IGFBP-3 were available only for pediatric patients pre-transplant and at regular intervals until several years post-HSCT. During the study period, three cross-calibrated assays for determining IGF-1 and IGFBP-3 levels were applied: RIA before 2008 as described by Juul et al. (24), Immulite 2000 IGF1/IGFBP3 (Siemens Healthcare Diagnostics, Tarrytown, NY, USA) from 2008 to 2013 and IDS-iSYS assays (Immunodiagnostic Systems LTD, Bolton, UK) based on chemiluminescence technology from 2013. These assays have all been validated and accredited by DANAK (25, 26). In all assays IGF-1 was dissociated from the binding proteins before quantification.

Serum levels were converted into age- and sex-adjusted SD-scores (SDS) calculated with normal ranges based on IGF-1 and IGFBP-3 measurements in > 5,000 healthy Danish children analyzed with the same three assays (RIA-method (24), Immulite-method (27, 28), and generalized additive models for location, scale and shape methodology for the iSYS-method).

### Inflammatory Parameters

According to our clinical routine levels of CRP were measured daily in 540 of the patients (99%) during hospitalization using Modular P module (Roche, Basel, Switzerland). Post-transplantation CRP_max_ was defined as the maximum CRP value from day +1 to day +21.

### Statistical Analyses

The IGF-I and IGFBP-3 levels were normally distributed. Age- and sex-adjusted SD-scores were applied to account for the natural differences related to sex and age. Two-tailed Student's *t*-test was used to assess the associations between SNPs and circulating IGF-1/IGFBP-3 levels under assumption of recessive inheritance mode where subjects who were homozygous for the minor allele were compared with the other genotypes, based on prior findings primarily stating a recessive inheritance mode for the included SNPs. rs35767 genotypes were compared by a dominant inheritance mode due to limited number of patients homozygous for minor allele and prior studies indicating dominant inheritance (29).

The association between CRP over time and SNP genotypes were analyzed using a mixed model with a compound symmetry covariance matrix and the significance was assessed with a Wald test with the degrees of freedom calculated using the Satterthwaite approximation.

The Mann-Whitney U-test was used when investigating the association between genotypes and CRP_max_. Multiple linear regression model was used when assessing effect of transplant-related risk factors as indicated under results. For conditioning regimen two groups were defined: high-intensity myeloablative conditioning (TBI ≥ 12 Gy or high-intensive chemotherapy) and low-intensity myeloablative conditioning (Fludarabine-based) (30).

Median follow-up time was calculated by the reverse Kaplan-Meier estimate (31). Kaplan–Meier estimates with log-rank tests were applied as an initial non-parametric analysis of probability of overall survival (OS), while cumulative incidences were estimated for risk of aGvHD and TRM to accommodate competing risks. Comparison of cumulative incidence was done using the Gray's test.

Linkage disequilibrium (LD) between SNPs was calculated as pairwise R-square correlation coefficients using the “genetics”-package (ver. 1.3.8.1.2) in R. Due to high linkage disequilibrium between two SNPs in *IGF1* (rs6220 and rs978458, LD = 0.89) and two SNPs in *IGFBP3* (rs2854744 and rs2854746, LD = 0.81) (Figure S2) only rs978458 and rs2854744 (chosen based on most consistent findings of association with circulating protein levels) were included in statistical analyses and function as tagging SNP for the excluded SNPs.

A two-sided *P*-value < 0.05 was considered significant without adjustment for multiple testing given the fact that the SNPs were already selected based on previously documented associations with levels of IGF-1/IGFBP-3 at Bonferroni corrected levels of statistical significance in large GWAS or candidate gene studies. All statistical analyses were performed using R statistical software version 3.6.1 (R Foundation for Statistical Computing, Vienna, Austria) and RStudio version 1.2.1335 (RStudio, Boston, MA).

### Ethics Statement

The study was approved by the ethics committee of the Capital Region of Denmark (H-15006001) and written informed consent was obtained from all patients and/or their legal guardians in accordance with the Declaration of Helsinki.

## Results

### IGF-1 and IGFBP-3 Levels

To confirm the impact of the selected SNPs on circulating protein levels in a HSCT setting we applied IGF-1 and IGFBP-3 serum measurements, which had been determined at pre-scheduled time intervals as part of the endocrinologic follow-up routine only at the pediatric HSCT unit and were available for 172 children. These children did not differ significantly from the other included children without IGF-1/IGFBP-3 measurements in terms of sex, age, diagnosis, donor type, conditioning regimen, graft type, pre-transplant Karnofsky score or sex-mismatch.

**Figure 1:**
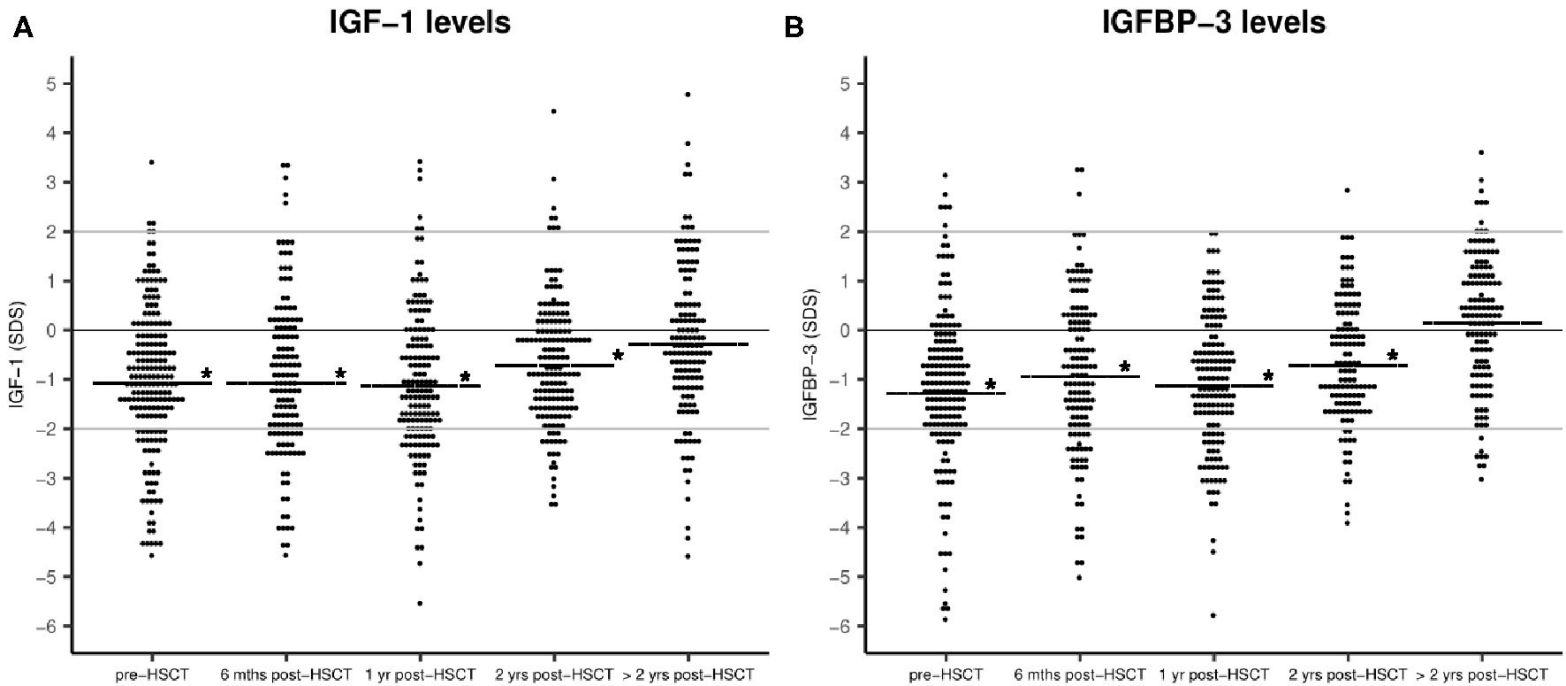
IGF-1 **(A)** and IGFBP-3 **(B)** plasma levels before and after HSCT as sex- and age-adjusted SD-scores. Horizontal line: mean IGF-1/IGFBP-3 SDS. Statistical evaluation indicates IGF-1 and IGFBP-3 levels compared with healthy population (0 SDS), *P < 0.001. No. of patients = 172.

From referral to transplant and until 2 years post-HSCT IGF-1 and IGFBP-3 levels were significantly reduced compared with healthy sex- and age-matched children (pre-HSCT: mean −1.06 SDS, 95% CI −1.3 to 0.8, *P* < 0.001 for IGF-1 and mean −1.2 SDS, 95% CI −1.5 to 1.0, *P* < 0.001 for IGFBP-3) (Figure 1). Pre-transplant levels were significantly lower for older children (−0.1 SDS per year, *P* < 0.001 (both IGF-1 and IGFBP-3 levels) but were not related to sex (*P* = 0.9) or diagnosis (malignant vs. non-malignant) (*P* = 0.6). Patients undergoing high-intensity myeloablative conditioning had generally lower IGF-1 and IGFBP-3 levels post-transplant (at 1-year post-HSCT: mean −1.2 vs. −0.67 SDS, *P* = 0.04 and mean −1.2 vs. −0.68 SDS, *P* = 0.03, respectively). Due to risk of mismatch between chronological age and delayed biological age we performed similar assessment of IGF-1/IGFBP-3 levels based on bone age-adjusted SD-scores, however with similar findings. We found no difference between IGF-1/IGFBP-3 levels at the timepoint of diagnosis and levels at referral to HSCT after chemotherapy treatment for 59 children with a malignant diagnosis for whom these measurements were available (data not shown).

### IGF-1 and IGFBP-3 Genotypes

Minor allele frequencies for included SNPs ranged between 14 and 38%. The genotype distributions correspond to reported gene frequencies in Caucasian cohorts by the NCBI dbSNP database and met the criteria for Hardy–Weinberg equilibrium for all SNPs (Table 2). No patient- or transplant-related characteristics (sex, age, diagnosis, conditioning regimen, donor type and graft type) were associated with any of the IGF1 genotypes in univariate analyses (all *P* > 0.08).

**Figure 2:**
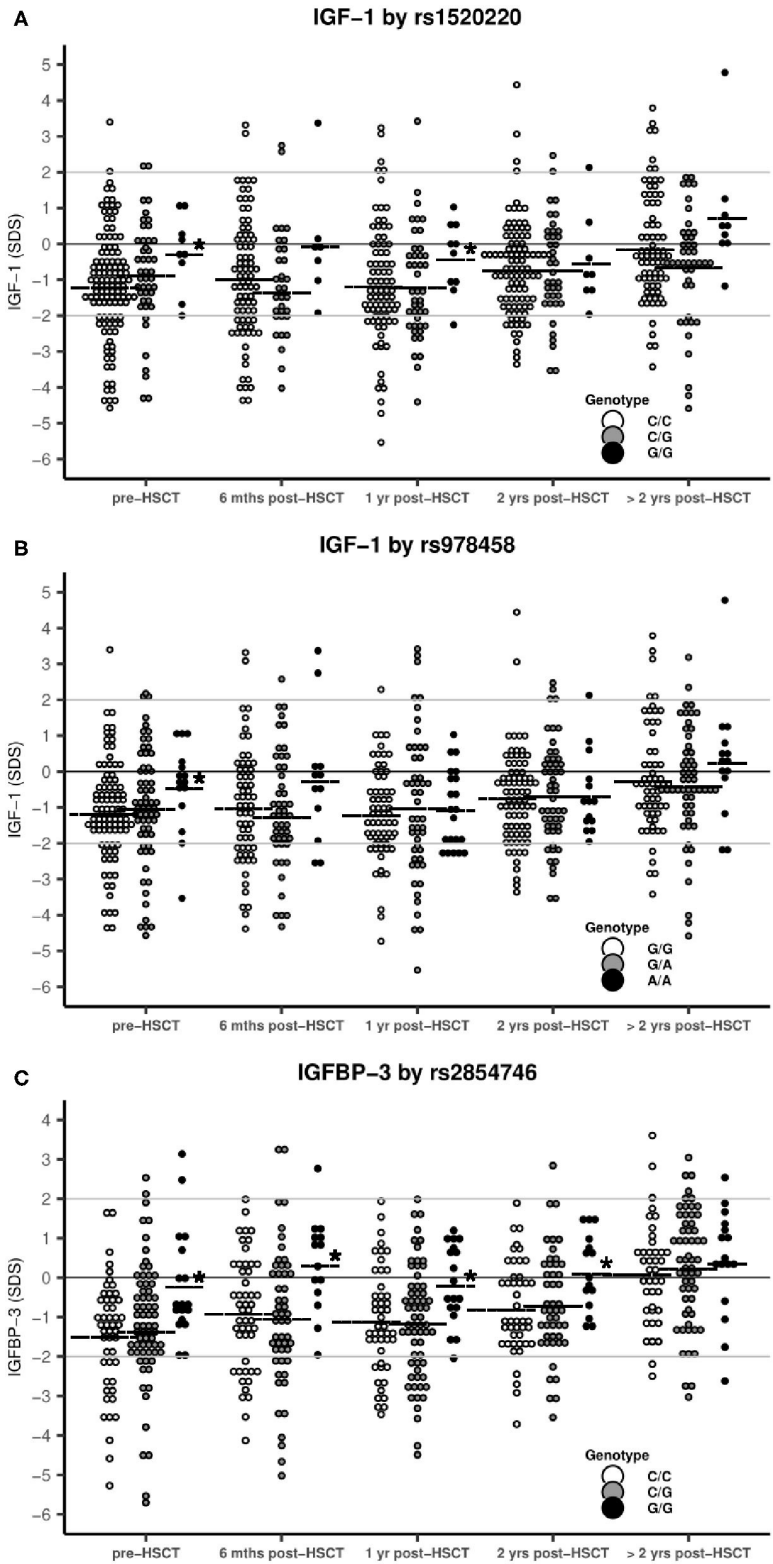
IGF-1 and IGFBP-3 levels stratified by SNP genotypes. Horizontal line: mean IGF-1/IGFBP-3 SDS. *statistical difference between genotypes. **(A)** rs1520220: Mean IGF-1 levels were significantly higher in patients with the G/G genotype pre-HSCT (*P* = 0.002) and at 1 year post-HSCT (*P* = 0.05) compared with the C/C+C/G genotypes (genotype distribution: C/C: *n* = 118, C/G: *n* = 45, G/G: *n* = 9). **(B)** rs978458: Mean IGF-1 levels were significantly higher in patients with the A/A genotype pre-HSCT (*P* = 0.007) compared with all other patients (genotype distribution: G/G: *n* = 93, G/A: *n* = 64, A/A: *n* = 15). **(C)** rs2854746: Mean IGF-1 levels were significantly higher in patients with the G/G genotype pre-HSCT (*P* = 0.002), at 6 months (*P* = 0.002), at 1 year (*P* = 0.004) and at 2 years post-HSCT (*P* = 0.006) compared with all other patients (genotype distribution: C/C: *n* = 59, C/G: *n* = 84, G/G: *n* = 22).

While we observed a significant reduction in IGF-1 and IGFBP-3 levels pre- and post-transplant for the overall pediatric study population, children homozygous for the high-producer minor alleles of rs1520220 and rs978458 *IGF1* SNPs or of rs2854746 *IGFBP3* had pre-transplant protein levels close to normal and significantly higher levels compared with the other genotypes (Table 2 and Figure 2), also after adjusting for age (rs1520220: *P* = 0.01, rs978458: *P* = 0.02 and rs854746: *P* = 0.01). The IGF-1/IGFBP-3 levels remained higher post-transplant for the rs1520220 and rs2854746 minor allele homozygous genotypes (Figures 2A,C), also after adjusting for potential post-transplant confounders (age, donor type, graft type, and conditioning regimen). None of the other SNPs were associated with circulating levels of their respective protein.

### SNPs and Systemic Inflammation

For the entire cohort (*N* = 543), median CRP level pre-HSCT was within normal range [median 4 mg/L; interquartile range (IQR) 1–12 mg/L (ref. < 10 mg/L)]. From day +4 to day +20 post-transplantation CRP increased significantly (*P* < 0.001 vs. day 0), peaking at day +10.

Homozygous carriers of the minor allele of rs1520220 or rs978458 in *IGF1* had significantly lower CRP_max_ during the early toxic phase (day +1 to +21 post-HSCT) than the other genotypes [rs1520220: median (IQR), 66 (28–134) vs. 102 (42–187) mg/L; *P* = 0.005 and rs978458: median (IQR), 53 (26–126) vs. 104 (43–188) mg/L; *P* < 0.001]. This was confirmed in a multiple linear regression model adjusting for age, conditioning regimen, and graft type (*P* = 0.04 and *P* = 0.02 for rs1520220 and rs978458, respectively), as these were found associated with levels of CRP_max_ in univariate analyses.

**Figure 3:**
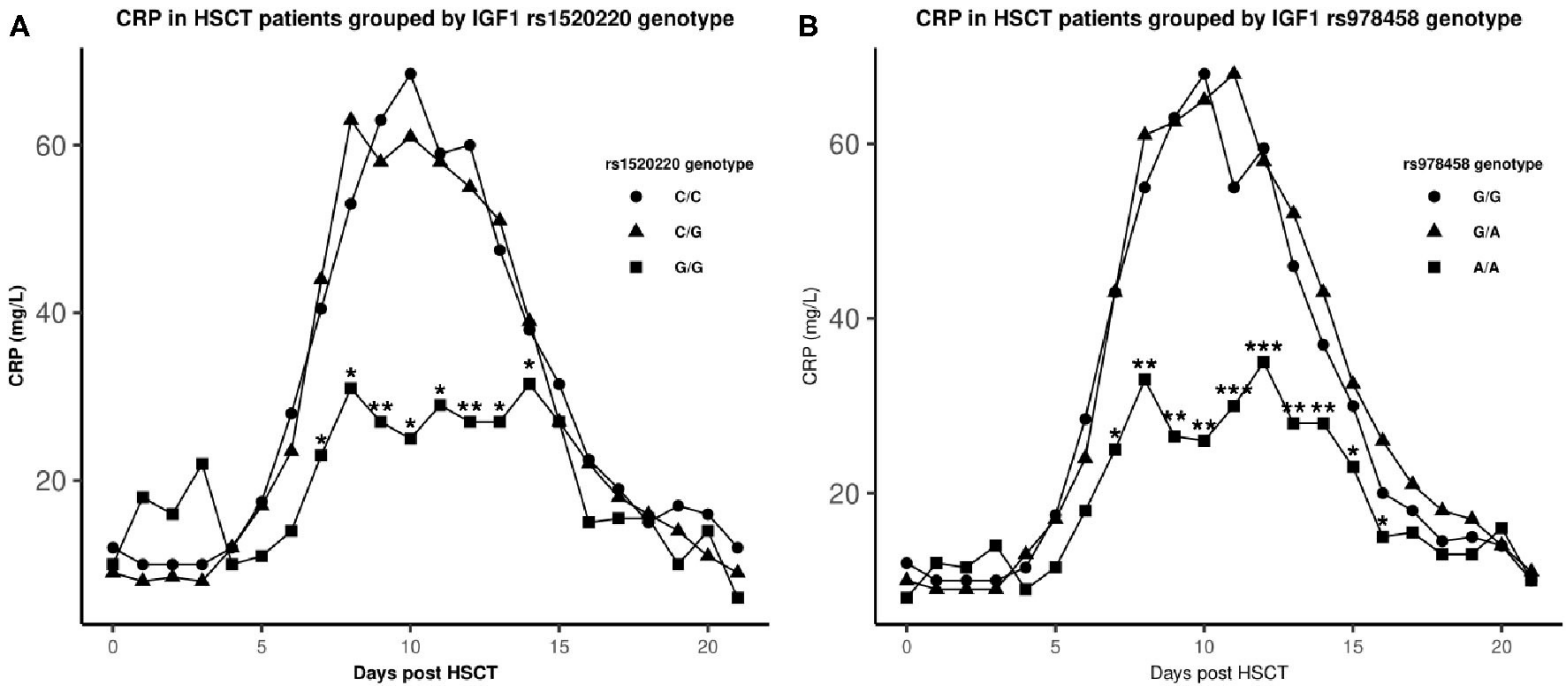
Median CRP levels after HSCT in genotype groups. ▴: homozygous major allele, •: heterozygous, ■: homozygous minor allele. *statistical difference in CRP levels between the homozygote minor allele genotype and the other genotypes (*P* ≤ 0.04). **P < 0.01, ***P < 0.001. Genotype distribution: rs1520220: C/C: *n* = 361, C/G: *n* = 160, G/G: *n* = 21 and rs978458: G/G: *n* = 297, G/A: *n* = 213, A/A: *n* = 33.

Additionally, regarding daily CRP through the first 3 weeks we could demonstrate lower daily CRP levels in homozygous carriers of the minor allele from day +7 to day +14 for rs1520220 (all *P* < 0.04), and from day +7 to day +16 for rs978458 (all *P* < 0.04) (Figure 3).

### SNP Genotypes and Clinical Outcomes

A total of 286 patients (52.7%) developed aGvHD within 90 days with onset at median day +23 (range +6 to +86) post-HSCT. Grades III–IV aGvHD were seen in 55 patients (10.1%). Homozygosity for the high-producer allele of rs978458 (*IGF1*) was associated with reduced risk of grade III–IV aGvHD in a univariate model with borderline significance (3.0% for the AA vs. 10.8% for the GG/GA genotype, *P* = 0.063). No associations between aGvHD and the other genotypes or between aGvHD at specific organ sites (gut, skin or liver) and any genotypes were observed.

**Figure 4:**
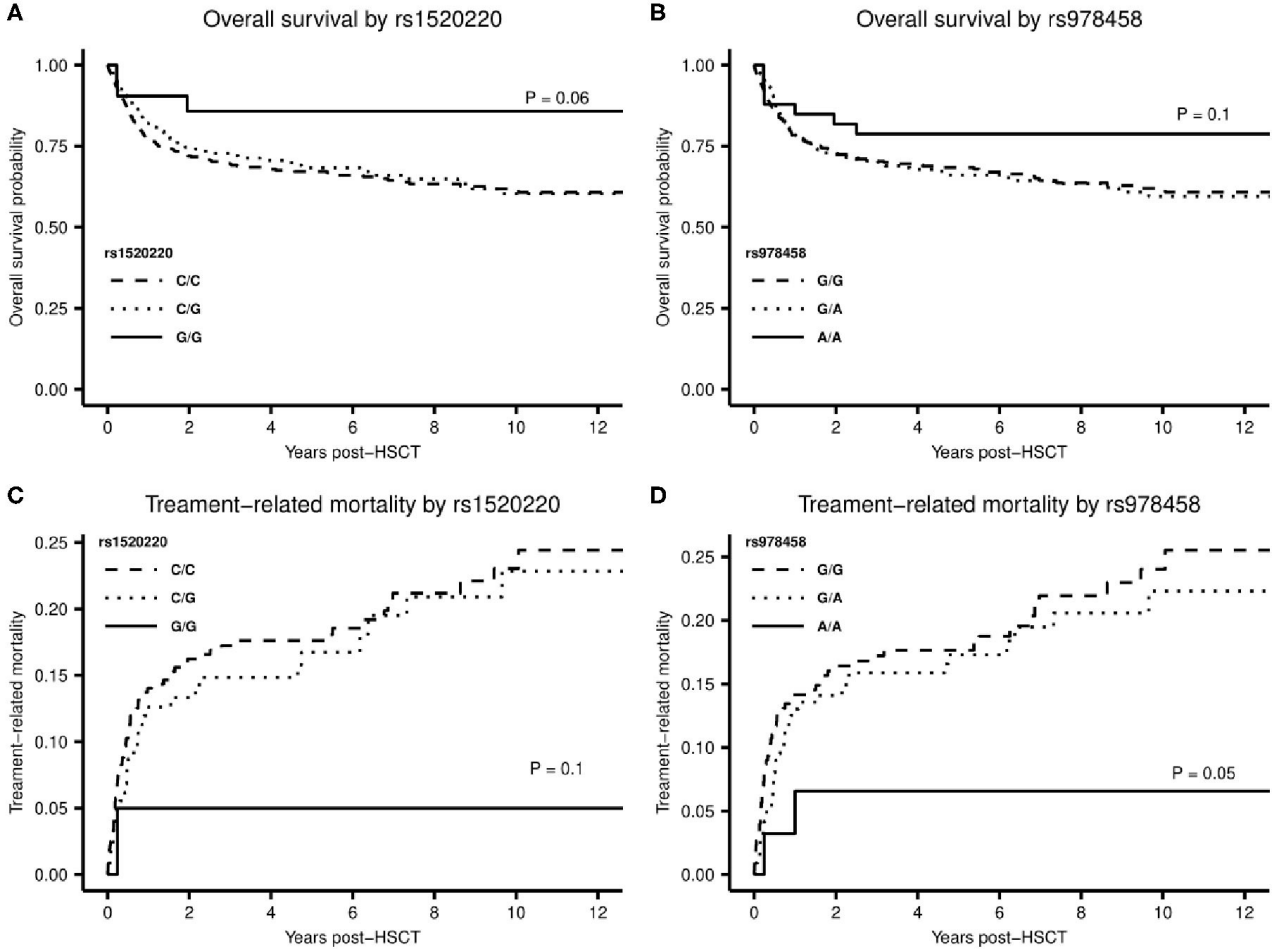
**(A,B)** Kaplan-Meier plot with log-rank test for difference in overall survival between the homozygote minor allele genotype and the other genotypes of rs1520220 **(A)**, *P* = 0.06 and rs978458 **(B)**, *P* = 0.1. **(C,D)** Cumulative incidence plot for treatment-related mortality stratified by genotypes of rs1520220 **(C)** and rs978458 **(D)**. **(C)** difference between the G/G genotype and the other genotypes (genotype distribution: C/C: *n* = 361, C/G: *n* = 160, G/G: *n* = 21), *P* = 0.1. **(D)** difference between the A/A genotype and the other genotypes (genotype distribution: G/G: *n* = 297, G/A: *n* = 213, A/A: *n* = 33), *P* = 0.05.

A total of 189 patients (35%) died within the follow-up time of median 8.0 years (range 2.0–14.3 years). Of patients with a malignant diagnosis, 90 patients (20.2%) died in remission. A Kaplan-Meier plot for OS and a cumulative incidence plot for TRM showed a tendency to superior OS and reduced risk of TRM for homozygous carriers of high-producer minor alleles for rs1520220 and rs978458 (Figure 4). This reached statistically significance for TRM for rs978458 (*P* = 0.050) and borderline significance for OS for rs1520220 (*P* = 0.060) (Figures 4A,D).

## Discussion

In this study we present evidence that IGF-1 high-producer genotypes are protective against an increased toxic inflammatory response to the cytotoxic treatment in patients undergoing HSCT.

IGF-1 is a well-known trophic factor for intestinal epithelial cells due to its mitogenic and anti-apoptotic effects under both physiological and pathophysiological conditions (18). In animal models, IGF-1 administration has been shown to limit mucositis, enhance mucosal repair and reduce bacterial translocation following irradiation or chemotherapy-induced damage (18, 19, 32). Thus, IGF-1 may limit systemic inflammation, induced as a result of barrier disruption (3), with potential impact on organ toxicity and transplant-related mortality (3–5, 7). Accordingly, our findings support the hypothesis that IGF-1 protects against a harmful inflammatory response by stabilizing the epithelial barrier in the gut.

Moreover, the observed association between high-producer alleles and reduced systemic inflammation may be explained by a more direct anti-inflammatory effect of IGF-1. In addition to a healing effect on epithelial cells, IGF-1 has been shown to attenuate both local and systemic inflammation by suppressing Th1 responses through mechanisms that involve modulation of pro-inflammatory cytokine signaling by macrophages and lymphocytes while promoting an Th2 response with release of anti-inflammatory cytokines by lymphocytes and monocytes in the gut (12, 17, 33). Notably, treatment with exogenous IGF-1 in combination with IGFBP-3 in mice and in children, and local IGF-1 gene therapy in rats, all with burn-induced inflammation, has been shown to prevent release of the pro-inflammatory cytokines IL-1β and tumor necrosis factor-α while increasing expression of IL-4 (14, 15, 17).

In accordance with our results, previous studies found endogenous IGF-1 and IGFBP-3 levels negatively associated with the degree of inflammation both in HSCT settings and in patients with inflammatory bowel disease. Further, in young male smokers and healthy middle-aged and elderly adults, low levels of IGF-1 were associated with chronic low-grade inflammation (16, 20, 34–36).

The finding of overall reduced IGF-1 levels in the children before chemotherapy treatment is in line with previous findings of low IGF-1 levels at diagnosis in children with acute lymphoblastic leukemia and in children with benign hematological diseases (37–39). The mechanism behind the observed reduction at this stage is not clear, but a severe catabolic state and modulation of the GH-IGF axis have been suggested (37, 39). The reduced IGF-1 levels seen up to 2 years post-transplant in our study most likely demonstrate a persistent influence of the toxic effects of the transplantation and conditioning. It is probable that this long persisting IGF-1 reduction will contribute to late effects, such as impaired growth, sarcopenia, endocrine disorders, metabolic syndrome and frailty seen in long-term survivors, and should be addressed in follow-up studies.

A difficulty in the interpretation of data on circulating IGF-1 is the lack of knowledge regarding to what degree a reduction in IGF-1 and IGFBP-3 plasma levels is caused by decreased production of IGF-1 and IGFBP-3 or increased consumption/turnover or translocation from the circulation to the tissues. Additionally, circulating IGF-1 is primarily regulated by GH and produced in the liver, whereas local production in the gut mucosa is also regulated by local factors including glucagon-like peptides 1 and 2 (40–42), the former recently found to increase significantly during the early toxic phase after high-dose chemotherapy in our recent study (43). Hence, plasma levels of IGF-1 may not properly reflect local IGF-1 concentrations and the bioavailability of IGF-1 for repair of the tissues.

However, studies of *IGF1* gene variation with impact on IGF-1 production, including local production, will complement previous investigations that were restricted to the study of plasma IGF-1 levels. The present study is, to our knowledge, the first to address association between systemic inflammation and genetic variants of the IGF-1 axis. By investigating the genetic variance in *IGF1* and *IGFBP3*, this study provides additional evidence for a protective effect of IGF-1 against tissue damage. It is likely that IGF-1 expressing cells in individuals homozygous for the minor allele of rs1520220 or rs978548 are able to produce higher amounts of IGF-1 when exposed to the various secretory stimuli resulting in better protection from chemotherapy-induced tissue damage.

The SNPs included in this study have been studied extensively in other clinical settings. The rs1520220 SNP was shown to be associated with increased risk of breast cancer in women carrying two copies of the minor allele (44) and of stomach cancer in a Japanese population (45). Eight large cohort studies have found the rs1520220 SNP to be associated with circulating IGF-1 levels (Figure S1). With our finding of rs978458 being associated with IGF-1 levels we confirmed the result of a large GWAS meta-analysis with 30,884 adults of European ancestry from 21 studies (46). It is important to note that the genotype distributions of all included SNPs in the present study were similar to those reported for healthy Caucasians (NCBI dbSNP) stating no association between the *IGF1*- and *IGFBP3* SNPs and the patients' pre-transplant diagnosis.

The precise molecular consequences of a SNP at rs1520220 and rs978458 in *IGF1* are unclear. Though, our observations indicate the presence of balanced polymorphisms where certain genotypes may be advantageous under certain circumstances, including tissue damage, as seen here, while, conversely, increasing the risk of fatal disease such as malignancies.

It could be speculated if treatment with IGF-1 in patients with the major allele of rs1520220 or rs978458 could limit early toxicities. Recombinant human (rh) IGF-1 has already been investigated as a treatment to improve outcome after intestinal epithelial damage, but electrolyte imbalances and hypoglycemia have complicated its use (47). However, a combination of rhIGF-1 and rhIGFBP-3 after burn injuries has shown attenuation of acute phase inflammation without unwanted side effects (17). Nonetheless, an important concern is the safety in neoplastic settings since IGF-1 is a mitogenic factor in erythropoiesis, granulopoiesis and lymphopoiesis (48). Moreover, considering that IGF receptors are expressed on most hematopoietic cells further studies including IGF-related gene polymorphisms of the donor are warranted to address the potential relevance as a supplement to HLA matching in donor selection (48). Further to this, future studies should explore the potential role of IGF-1treatment in limiting the toxicities of HSCT. In addition, this study give weight to the notion that epithelial protection by growth factors is of importance during HSCT. Other growth factors with similar trophic effects may be considered, including glucagon-like peptide 1 and 2.

A strength of the study is the homogeneity of the study population limited to one ethnic group seen in a population-based setting and a relatively large sample size for a study of HSCT patients. Additionally, the present study was restricted to SNPs previously confirmed to impact protein levels in healthy individuals, thereby limiting the risk of false positive findings and loss of power due to extensive correction for multiple testing which is often an issue in studies using extensive genotyping (49).

Our study has limitations related to its retrospective design and lack of its own validation cohort. Further, the relatively low frequency of patients being homozygous for the IGF-1 high-producer alleles (4–6%) leads to limitations in terms of statistical power to conclusively study the effects on the clinical outcomes. Accordingly, conclusive stratified analysis and multivariable analyses related to survival could not be performed. Of note, however, IGF-1 and IGFBP-3 were both unrelated to any patients- and transplant-related risk factors.

To conclude, this study is, to our knowledge, the first to investigate genetic variations in the *IGF1* gene in the setting of HSCT. We found that genotypes causing high IGF-1 plasma levels are associated with reduced systemic inflammation and improved survival, most likely reflecting a tissue protecting effect of IGF-1 against the cytotoxic treatment. Accordingly, this study lends support to the notion that trophic growth factors influence the huge variability in how patients respond to the harmful effects of this treatment, pointing to new directions in the search for effective strategies to limit the toxicities related to cancer treatment and HSCT.

## Data Availability Statement

The data that support the findings of this study are available on request from the corresponding author. The data are not publicly available due to ethical restrictions of data containing information that could compromise the privacy of research participants.

## Ethics Statement

The studies involving human participants were reviewed and approved by the ethics committee of the Capital Region of Denmark (H-15006001). Written informed consent to participate in this study was provided by the participants' legal guardian/next of kin.

## Author Contributions

ME contributed to data collection, performed statistical analyses, interpretation, and drafted the manuscript. CE and AJ contributed to project design and data interpretation and performed laboratory analyses. CH and HS established sample collection design and contributed to sample collection. KM designed the project, established the collaboration, and contributed to data interpretation. All authors contributed to the article and approved the submitted version and critically revised the manuscript.

## Conflict of Interest

The authors declare that the research was conducted in the absence of any commercial or financial relationships that could be construed as a potential conflict of interest.
