# Supplementary material for: Insulin-Like Growth Factor Gene Polymorphisms Predict Clinical Course in Allogeneic Hematopoietic Stem Cell Transplantation
Source: Front Immunol. 2020 Jul 24;11:1646. doi: 10.3389/fimmu.2020.01646 (PMC7393983; doi:10.3389/fimmu.2020.01646)
Supplement: Supplementary file 1 [file Data_Sheet_1.docx]

**
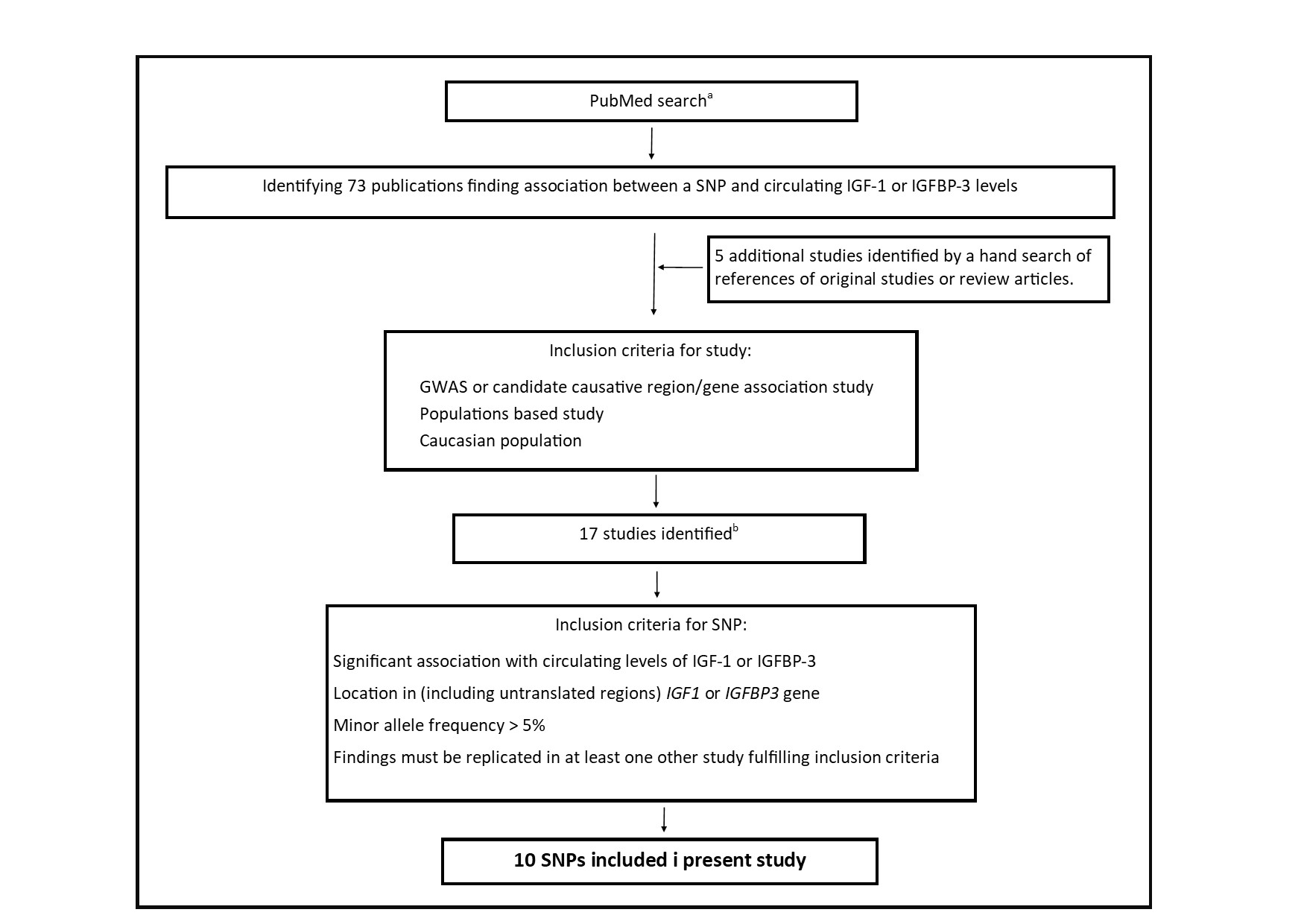
**

**Figure S1:** SNP selection flowchart

^a^Search term: (”Genetic variation” OR ”polymorphism” OR snp) AND (IGF1 OR “IGF-1” OR ”insulin-like growth factor 1”) AND (“serum levels” OR “serum level” OR “plasma levels” OR “plasma level” OR “circulating levels” OR “circulating level”)

^b^References to included studies:

Aloisio AAD, Schroeder JC, North KE, Poole C, Suzanne L, Travlos GS, Baird DD. IGF-I and IGFBP-3 polymorphisms in relation to circulating levels among African American and Caucasian women. *Cancer Epidemiol Biomarkers Prev* 2010;18:954–66.

Al-Zahrani A, Sandhu MS, Luben RN, Thompson D, Baynes C, Pooley KA, Luccarini C, Munday H, Perkins B, Smith P, Pharoah PDP, Wareham NJ, et al. IGF1 and IGFBP3 tagging polymorphisms are associated with circulating levels of IGF1, IGFBP3 and risk of breast cancer. *Hum Mol Genet* 2006;15:1–10.

Biong M, Gram IT, Brill I, Johansen F, Solvang HK, Alnaes GIG, Fagerheim T, Bremnes Y, Chanock SJ, Burdett L, Yeager M, Ursin G, et al. Genotypes and haplotypes in the insulin-like growth factors, their receptors and binding proteins in relation to plasma metabolic levels and mammographic density. *BMC Med Genomics* 2010;3:no.9.

Canzian F, McKay JD, Cleveland RJ, Dossus L, Biessy C, Rinaldi S, Landi S, Boillot C, Monnier S, Chajès V, Clavel-Chapelon F, Téhard B, et al. Polymorphisms of genes coding for insulin-like growth factor 1 and its major binding proteins, circulating levels of IGF-I and IGFBP-3 and breast cancer risk: results from the EPIC study. *Br J Cancer* 2006;94:299–307.

Diorio C, Brisson J, Bérubé S, Pollak M. Genetic polymorphisms involved in insulin-like growth factor (IGF) pathway in relation to mammographic breast density and IGF levels. *Cancer Epidemiol Biomarkers Prev* 2008;17:880–8.

Gu F, Schumacher FR, Canzian F, Allen NE, Albanes D, Berg CD, Berndt SI, Boeing H, Bueno-de-Mesquita HB, Buring JE, Chabbert-Buffet N, Chanock SJ, et al. Eighteen insulin-like growth factor pathway genes, circulating levels of IGF-I and its binding protein, and risk of prostate and breast cancer. *Cancer Epidemiol Biomarkers Prev* 2010;19:2877–87.

Johansson M, McKay JD, Wiklund F, Rinaldi S, Verheus M, Van Gils CH, Hallmans G, Bälter K, Adami HO, Grönberg H, Stattin P, Kaaks R. Implications for prostate cancer of insulin-like growth factor-I (IGF-I) genetic variation and circulating IGF-I levels. *J Clin Endocrinol Metab* 2007;92:4820–6.

Ollberding NJ, Cheng I, Wilkens LR, Henderson BE, Pollak MN, Kolonel LN, Le Marchand L. Genetic Variants, Prediagnostic Circulating Levels of Insulin-like Growth Factors, Insulin, and Glucose and the Risk of Colorectal Cancer: The Multiethnic Cohort Study. *Cancer Epidemiol Biomarkers Prev* 2012;21:810–20.

Palles C, Johnson N, Coupland B, Taylor C, Carvajal J, Holly J, Fentiman IS, dos Santos Silva I, Ashworth A, Peto J, Fletcher O. Identification of genetic variants that influence circulating IGF1 levels: A targeted search strategy. *Hum Mol Genet* 2008;17:1457–64.

Patel A V., Cheng I, Canzian F, Le Marchand L, Thun MJ, Berg CD, Buring J, Calle EE, Chanock S, Clavel-Chapelon F, Cox DG, Dorronsoro M, et al. IGF-1, IGFBP-1 and IGFBP-3 polymorphisms predict circulating IGF levels but not breast cancer risk: Findings from the breast and prostate cancer cohort consortium (BPC3). *PLoS One* 2008;3.

Ricketts SL, Rensing KL, Holly JM, Chen L, Young EH, Luben R, Ashford S, Song K, Yuan X, Dehghan A, Wright BJ, Waterworth DM, et al. Prospective study of insulin-like growth factor-I, insulinlike growth factor-binding protein 3, genetic variants in the IGF1 and IGFBP3 genes and risk of coronary artery disease. *Int J Mol Epidemiol Genet* 2011;2:261–85.

Schumacher FR, Cheng I, Freedman ML, Mucci L, Allen NE, Pollak MN, Hayes RB, Stram DO, Canzian F, Henderson BE, Hunter DJ, Virtamo J, et al. A comprehensive analysis of common IGF1, IGFBP1 and IGFBP3 genetic variation with prospective IGF-I and IGFBP-3 blood levels and prostate cancer risk among Caucasians. *Hum Mol Genet* 2010;19:3089–101.

Smith TJ. Insulin-like growth factor-I regulation of immune function: A potential therapeutic target in autoimmune diseases? *Pharmacol Rev* 2010;62:199–236.

SU X. Genetic Variation and Circulating Levels of IGF-I and IGFBP-3 in Relation to Risk of Proliferative Benign Breast Disease. *Int J Cancer* 2010;23:1–7.

Teumer A, Qi Q, Nethander M, Aschard H, Bandinelli S, Beekman M, Berndt SI, Bidlingmaier M, Broer L, Cappola A, Ceda GP, Chanock S, et al. Genomewide meta-analysis identifies loci associated with IGF-I and IGFBP-3 levels with impact on age-related traits. *Aging Cell* 2016;15:811–24.

Verheus M, McKay JD, Kaaks R, Canzian F, Biessy C, Johansson M, Grobbee DE, Peeters PHM, van Gils CH. Common genetic variation in the IGF-1 gene, serum IGF-I levels and breast density. *Breast Cancer Res Treat* 2008;112:109–22.

Åberg ND, Olsson S, Åberg D, Jood K, Stanne TM, Nilsson M, Blomstrand C, Svensson J, Isgaard J, Jern C. Genetic variation at the IGF1 locus shows association with post-stroke outcome and to circulating IGF1. *Eur J Endocrinol* 2013;169:759–65.


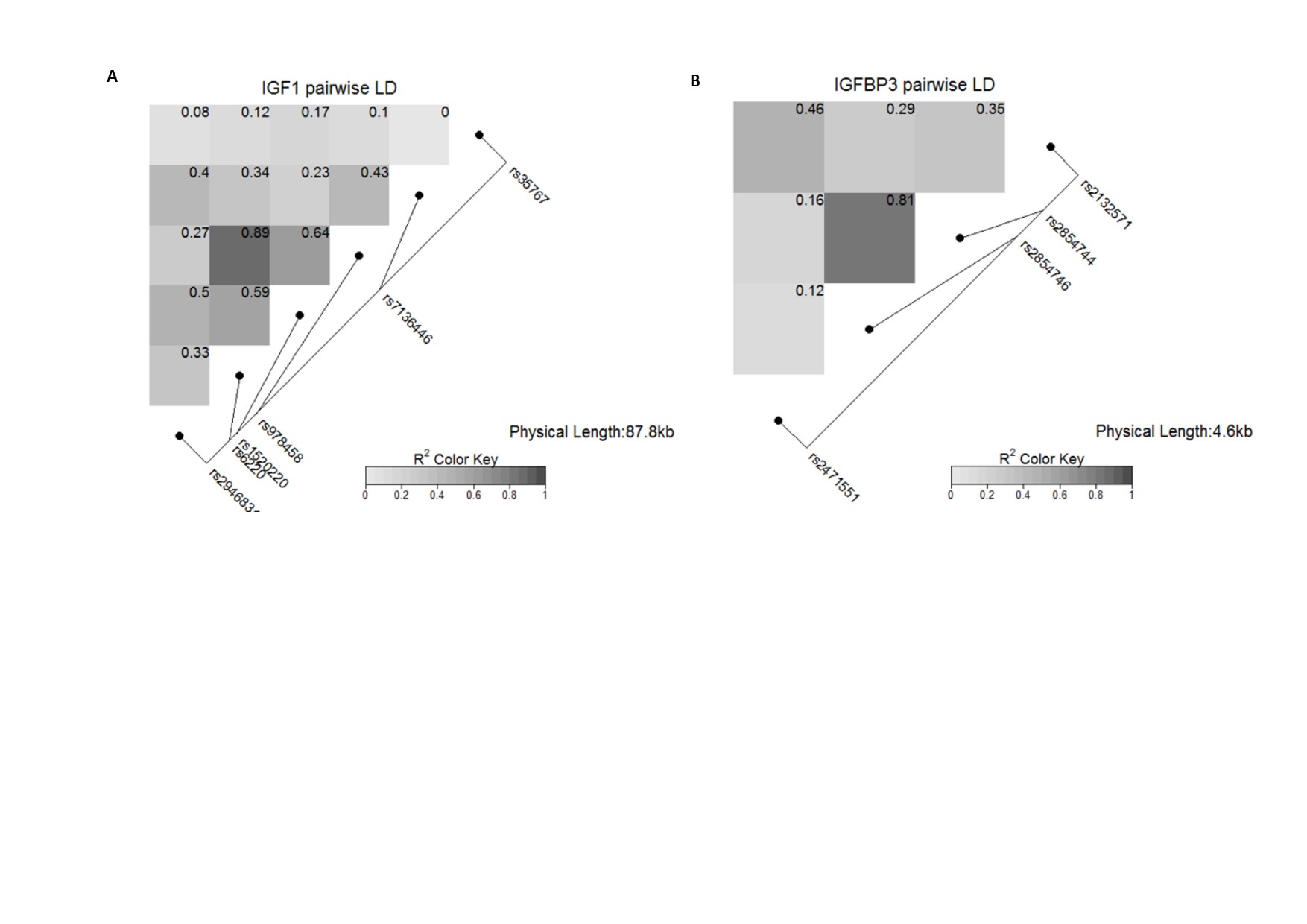


**Figure S2: (A)** Graphical representation of linkage disequilibrium (LD) of SNPs of *IGF1*. **(B)** Graphical representation of LD of SNPs of *IGFBP3*. The lines represent SNPs and physical distances among them. Numbers within squares are pairwise R^2^ values. The colour code shows confidence boundaries of LD estimations: darkest grey shows evidence of LD.
